# 

**Published:** 1915-01-16

**Authors:** 


### CONTENTS.

PAGE

**Some Influences of the Darkened Streets** ... 343

**The Reconstruction of the Metropolitan Asylums Board** ... ... ... ... ... 344

**Hospital and Institutional News** ... ... ... 345

The King and Queen at Brighton—Hospital Church Dedicated—The Master of the Rotunda Hospital's Resignation—The Special Hospital for Officers—A Vigorous Policy for Hertford County Hospital—How to Ensure the Appeal's Success—Ambulance Construction—The Education of Defective Children—A House Surgeon's Confession in a Coroner's Court—Insurance Against Damage by Hostile Aircraft—The Florence Nightingale Statue—Limited Companies as Hospital Subscribers—Consideration for Patients' Relatives — "Where are the Nine?"—A Medical Superintendent's Salary—Sculcoates Guardians' Temporary Expedient—The Red Cross in Cornwall—Registration of Belgian Medical Practitioners—The New Ulster Volunteer Force Hospital—The Scientific Press Diaries—The Stay of the Wounded in Hospital—A Hospital the Victim of a Dispute—Doctors' Gift to a Military Hospital—Two Doctors Poisoned—From Nurse to General Practitioner—Anti-typhoid Inoculation in America—This Week's Drug Market—To Our Readers.

PAGE

**Impressions from a Field Ambulance** ... ... 351

Tyres, Equipment, and the Health of an Army.

**Doctors' Wills** ... ... ... ... ... ... 352

**Medico-Psychology** ... ... ... ... ... 353

The Hallucinations of Hearing.

**The Involution of the Uterus and its Influence on Practical Therapy** ... ... 354

**Round the World** ... ... ... ... ... 355

Fellow-Workers and the Roll of Honour.

**Hospital Account Keeping and the War** ... 356

The Allocation of War Expenditure.

**Bleaching and Disinfecting by the Aid of Electrolysis** ... ... ... ... .. 357

Experiments at Bexley Mental Hospital.

**Buildings Contemplated** ... ... ... ... 358

**Practical Points in Hospital Matters** ... ... 359

An Improved Type of Motor Ambulance.

**Legal Notes for Institutional Workers** ... 360

[See over.]

### CONTENTS—(*continued*).

| | PAGE |
|---|---|
| **A Medical Officer Imprisoned in Germany** ... | 360 |
| **The Department of Modern Nursing** ... ... | 361 |
| VII.—The Middlesex Hospital. | |
| **The Editor's Letter-Box** ... ... ... ... | 363 |
| Boots for Belgian Wounded: Thanks from Worcester. | |
| **Medical Appointments** ... ... ... ... | 363 |
| **Tuberculosis Treatment in Middlesex** ... ... | 363 |
| **Gifts and Bequests** ... ... ... ... ... | 363 |
| **Obituary** ... ... ... ... ... ... ... | 363 |
| **Art Stamps for Charitable Purposes** ... ... | 364 |
| **The Book Market** ... ... ... ... ... ... | 364 |
| **The Institutional Worker** ... ... (Suppl.) | 1 |
| Answers to December Question: Contributions from Out-patients. | |
| A Nominal Fee for Registration or Attendance. | |
| Success Dependent on the Sister in Charge. | |
| Question for January. | |
| Questions Invited. | |
| The Sick and in Need. | |
| Employment and Training. | |
| Practical Matters. | |

